# The identification of novel missense variant in *ChAT* gene in a patient with gestational diabetes denotes plausible genetic association

**DOI:** 10.1515/med-2025-1225

**Published:** 2025-07-17

**Authors:** Oluwafemi G. Oluwole, Afolake Arowolo, Ezekiel Musa, Naomi Levitt, Mushi Matjila

**Affiliations:** Division of Human Genetics, Institute of Infectious Diseases and Molecular Medicine, University of Cape Town, PMB 7700, Observatory, Cape Town, South Africa; Biomedical Research Centre, Nuffield Department of Medicine, Wellcome Centre for Human Genetics, University of Oxford, Oxford, United Kingdom; Institute of Primate Research, Nairobi, Kenya; Biomedical Research and Innovation Platform (BRIP), South African Medical Research Council, Cape Town, South Africa; Department of Medicine, University of Cape Town, Cape Town, South Africa; Department of Internal Medicine, Kaduna State University, Kaduna, Nigeria; Department of Obstetrics and Gynaecology, University of Cape Town, Cape Town, South Africa

**Keywords:** gestational diabetes mellitus, placenta, *ChAT*, choline, genotype, variants

## Abstract

**Introduction:**

Gestational diabetes mellitus (GDM), the most common metabolic complication of pregnancy, is associated with a 50% increase in subsequent risk for type 2 diabetes. There is increasing interest in identifying biomarkers that may facilitate the stratification of subsequent type 2 diabetes risk among women with GDM. In this study, we considered the choline acetyltransferase (*ChAT*) gene. CHAT plays a critical role in acetylcholine synthesis and regulates insulin secretion from the pancreatic islet to maintain glucose homeostasis.

**Methods:**

We screened for deleterious variants in the *ChAT* gene in 12 GDM patients and 10 ethnically matched controls from a South African cohort. We isolated DNA from the placental samples of these patients and performed DNA sequencing of the protein-coding region of the *ChAT* gene. Sequence alignments and variant annotations were done using UGENE software and Ensembl VEP.

**Results:**

A novel heterozygous missense variant in exon 8 of the *ChAT* gene was identified. The plausible phenotypic impact of the variant *ChAT* (NM_020549.5):c.1213C>G (p.Leu405Val) can be explained by haploinsufficiency, changing protein activities, strong transcription activity, and epigenetic repression activities of the variant. Also, structurally, the variant is located 18bp in-frame to a stop-gained variant (p.Gly411Ter). The RegulomeDB DNase expression data clearly show the identified variant in a peak expression in the spleen and placenta. This observation corroborates that the *ChAT* gene may play an essential role in GDM.

**Conclusion:**

Taken together, the metric scores for this variant show that it could affect the functions of the gene, but more functional studies are necessary to validate these effects. Consequently, this study sets the stage for the future screening of a larger cohort and functional validation of deleterious variants to underpin the *ChAT* gene and GDM association.

## 1 Introduction

Gestational diabetes mellitus (GDM) [ICD-10 O24.429] is a metabolic disorder characterised by hyperglycaemia and insulin resistance that develops during pregnancy. GDM affects approximately 17 million pregnancies worldwide, with increasing prevalence owing primarily to the surge in obesity and advanced maternal age [1]. GDM occurs when certain placental hormones prevent the pancreas’s beta cells from effectively utilising or producing insulin, causing insulin resistance [2,3]. Despite being the most life-threatening condition that has resulted in the deaths of numerous pregnant women and foetuses, there is no definitive and universally applied genetic predisposing screening method for GDM. This fundamental limitation makes it very challenging to critically appraise the actual occurrence of this condition and associated complications [2–4].

GDM is a complex disorder, and its pathological mechanisms have primarily been investigated in biological interaction studies, which include genetic, epigenetic, and environmental factors [4]. However, the existing genetic studies, including GWAS of GDM, are restricted to a few from East Asia. About 12 single-nucleotide polymorphisms (SNPs) have been associated with GDM [5]; notably, *the Melatonin Receptor 1B gene* (*MTNR1B*) variants rs10830963 and rs1387153, which are strongly associated with GDM mainly in this population [4]. This variant has previously been reported to result in the increased expression of *MTNR1B*, correlated with high fasting plasma glucose and insulin levels and resistance. In addition, potassium voltage-gated channel subfamily Q member 1 (*KCNQ1*) is a gene implicated in insulin secretion [6]. For instance, SNPs rs 2074196 and rs 2237892 are associated with the risk of GDM in Korean women [4], while rs 2237892 and rs 2237895 were correlated with decreased insulinogenic index when GDM was diagnosed. GDM studies in Indian women identified insertion and deletion of mitochondrial tRNA genes associated with GDM, and SNPs identified in the *Potassium Inwardly Rectifying Channel Subfamily J Member 11* (*KCJN11*) and the *Growth factor receptor-bound protein 14* (*GRB14*, which encodes a protein that interacts with insulin and insulin-like growth-factor receptors) genes were also associated with GDM and type 2 diabetes (T2D) [5,7]. These studies contributed to the knowledge of GDM by suggesting that genetic polymorphism in several genes might play some roles in GDM, specifically impaired physiological functions related to insulin resistance and secretion [4]. Nonetheless, in some cases, like the studies on Indian women, the direction of the effect of the SNPs differed; some T2D risk variants were indicative of being protective against GDM in the women [5]. However, no such studies have investigated the role of gene mutations or genetic contributions to GDM in the African population. Studies have shown a relationship between GDM, choline synthesis, and metabolism [8–10].

Pregnancy is associated with a higher demand for choline intake due to accelerated one-carbon metabolism and the synthesis of new membranes as cells undergo division [8–12]. GDM causes a pronounced reduction of choline, suggesting that genes and receptors involved in choline synthesis and metabolism should be studied for potential aetiologic roles [8–12]. The *human Choline acetyltransferase* (*ChAT*) gene encodes the *ChAT* enzyme that catalyses the neurotransmitter acetylcholine (ACh) biosynthesis from acetyl-CoA and choline at cholinergic synapses. Diseases associated with *ChAT* include myasthenic syndrome, dementia, and Parkinson’s disease [4,13,14]. The cholinergic system has been strongly related to the causes of GDM. A study revealed that ACh-induced vasodilatation in both hands and feet was lower in women with previous GDM than in the control group [15,16]. The *ChAT/ACh*-associated pathways transmit ACh across chemical synapses and glycerophospholipid biosynthesis [17].

ACh, the primary neurotransmitter of the parasympathetic nervous system, can enhance glucose-stimulated insulin secretion (GSIS) from pancreatic beta-cells, leading to pancreatic beta-cell dysfunction or the delayed response of the beta cells to the rising glycaemic levels and marked insulin resistance secondary to placental hormonal release [4,18,19]. Additionally, since ACh regulates insulin secretion from the pancreas, it is crucial for glucose homeostasis [19]. How choline protein regulates insulin production is not fully understood. However, the earliest studies in these areas have provided evidence to leverage and investigate various aspects. For example, the study by Fishwick and Rylett used differentiated SH-SY5Y cells stably expressing choline proteins to study the effect of insulin signalling on choline activity and function. This study found that acute exposure of depolarised cells to insulin is coupled to transiently increased levels of choline proteins at the cell surface, which were attenuated by chronic insulin exposure. This model accurately depicts what usually happens during gestational diabetes, where irregularities occur in the cell’s exposure to insulin. Taken together, the rationale for this study is to determine the plausible roles of the *ChAT* gene in the pathogenicity of gestational diabetes. Given that there are literature reports that the ChAT gene is physiologically involved in insulin regulation [19]. In essence, individuals with pathogenic variants in one or both alleles of this gene who are diagnosed with gestational diabetes should be investigated further for pathophysiology and genetic diagnosis. Hence, this study examined the *ChAT* gene for pathogenic variants in a South African GDM women cohort.

## 2 Methods

### 2.1 Ethical approval, inclusion criteria, study participants, sample collections, and processing

This study received ethical clearance and approval from the Human Research Ethics Committee of the Faculty of Health Sciences, University of Cape Town (HREC: 463/2018). The study participants gave written consent. The participants were pregnant women who attended Groote Schuur and Mowbray Maternity Hospitals in Cape Town, South Africa. Therefore, the inclusion criteria for the study participants are (1) the patient is a female and pregnant, (2) the patient is fully diagnosed with GDM by a doctor, (3) the patient has no underlying severe conditions or has been diagnosed with diabetes type 1 or type 2, and (4) the control has no underlying condition, no GDM, or living with diabetes; furthermore, using an approximate ratio of 1:1, assuming that only one causal genetic marker in the *ChAT* gene is present in the case but absent in control. In all, the study participants consisted of 12 women diagnosed with GDM and 10 healthy, ethnically matched pregnant women as controls.

The placental tissue samples from these participants were collected post-delivery. DNA was extracted from these samples using the Genomic DNA Purification Kit from ThermoFisher Scientific according to the kit’s manufacturer’s protocol with some modifications. About 30 mg of flash-frozen placenta samples were finely diced and transferred into 2 mL containing 2 × 1.4 mm ceramic beads (Omni International) and the kit’s lysis buffer. This step was followed by sample homogenisation (4 × 30 s cycle) on a Beadruptor (Omni International) at 4°C, to which 200 µL of Tris-EDTA buffer pH 8.0 was added to the lysate, and the sample homogenate was incubated at 65°C for 10 min with mild agitation. After incubation, 600 µL of chloroform was used to extract the DNA into the aqueous phase of the lysate after centrifuging at 10,000 rpm for 2 min. DNA was precipitated from this aqueous phase using 800 µL of the kit’s 1× precipitation solution (diluted from a 10× concentrate with deionised water) and centrifuged at 10,000 rpm for 2 min after prior mixing by several inversions at room temperature for 2 min. The supernatant was then carefully aspirated, and precipitated DNA was dissolved in 100 µL of NaCl supplied with the kit and gently mixed. The RNA was digested using 0.2 mg/mL RNAse A (#EN0531, ThermoFisher Scientific) at 37°C for 10 min. The RNAse-free DNA was then re-precipitated with 300 µL of ice-cold ethanol for 10 min at −20°C and centrifuged at 10,000 rpm for 4 min. The supernatant was removed, the pellet containing the DNA was washed once with 70% ethanol, and the DNA was dissolved in sterile deionised water by gentle vortexing. The purified DNA from each sample was quantified using the Biodrop nucleic acid quantifier (Biochrom, UK; Table S1) and stored at −20°C. For the screening and genotyping assay, the concentrated DNA was diluted to a working concentration of 50–100 ng/μL with SABAX H_2_O (Adcock Ingram, South Africa) and measured using a nanodrop.

### 2.2 Screening the *ChAT* gene coding sequences in patients and controls

The primers for the polymerase chain reaction (PCR) and the sequencing primers for sequencing the coding regions of the gene were designed with Primer3Plus (https://www.bioinformatics.nl/cgi-bin/primer3plus/primer3plus.cgi), IDT Oligo Analyser 3.1 (https://eu.idtdna.com/pages/tools/oligoanalyzer), and NCBI Primer Blast (https://www.ncbi.nlm.nih.gov/tools/primer-blast/). The human *CHAT* gene was amplified using PCR with the primer set below:

Forward primers: 5′ AAATGCTGAGCTAGGGGCAG-3′

Reverse primers: 5′ GAATCTACCCGGAGCGCTTC-3′

The sequencing primers targeting exons 2, 4, 6, 8, 10, and 12 of the *CHAT* gene were used for genotyping and screening for the disease-associated variants in the *ChAT* gene (Table S2). The PCR was performed using the optimised conditions for each primer pair to amplify the coding region of *the ChAT* gene in the 1 µL DNA (150 ng/μL) using the *GoTaq* ^®^ (Promega) protocol and reagents. The amplified PCR products were cleaned up, and the sequencing reaction was performed with a BigDye^®^ Terminator kit (Applied Biosystems), and capillary electrophoresis was performed using the ABI PRISM^®^ 3130xl Genetic Analyser (Applied Biosystems by ThermoFisher Scientific).

### 2.3 Analyses of sequenced data

The QC report of the sequenced samples (Table S3) was analysed, and we removed the sequences with low signal-to-strength values. The sequenced alignment and analyses were performed using the UGENE v.40 and Bioedit software. A two-step analysis was used. The first step focused on the clinically relevant variants in the *ChAT* gene that have been identified and catalogued in ClinVar and OMIM databases (Table S2). The second step focused on identifying novel (AF = 0) and rare variants in *ChAT*. Variant annotations were performed with VEP Ensembl (https://www.ensembl.org/index.html) and Varsome database (https://varsome.com/), as well as the variant annotation tools SIFT (https://sift.bii.a-star.edu.sg/), CADD (https://cadd.gs.washington.edu/score), and PolyPhen2 (http://genetics.bwh.harvard.edu/pph2/). To determine the rare allele frequency for each variant, we queried the variant in the gnomAD database, which currently has 500,000 exomes and whole genomes. Furthermore, the impact of the variant identified in this study was annotated by querying the RegulomeDB (https://www.regulomedb.org) database for epigenetic, gene regulatory, and tissue-specific functions. The *ChAT* gene profiling for enrichment terms, protein–protein interactions, and gene constraint status was determined with STRING and GeneMania.


**Informed consent:** The study participants gave written consent to participate, which also included publishing de-identified data from this study.
**Ethical approval:** The Human Research Ethics Committee of the Faculty of Health Sciences, University of Cape Town, approved this study (HREC: 463/2018).

## 3 Results

**Figure 1:**
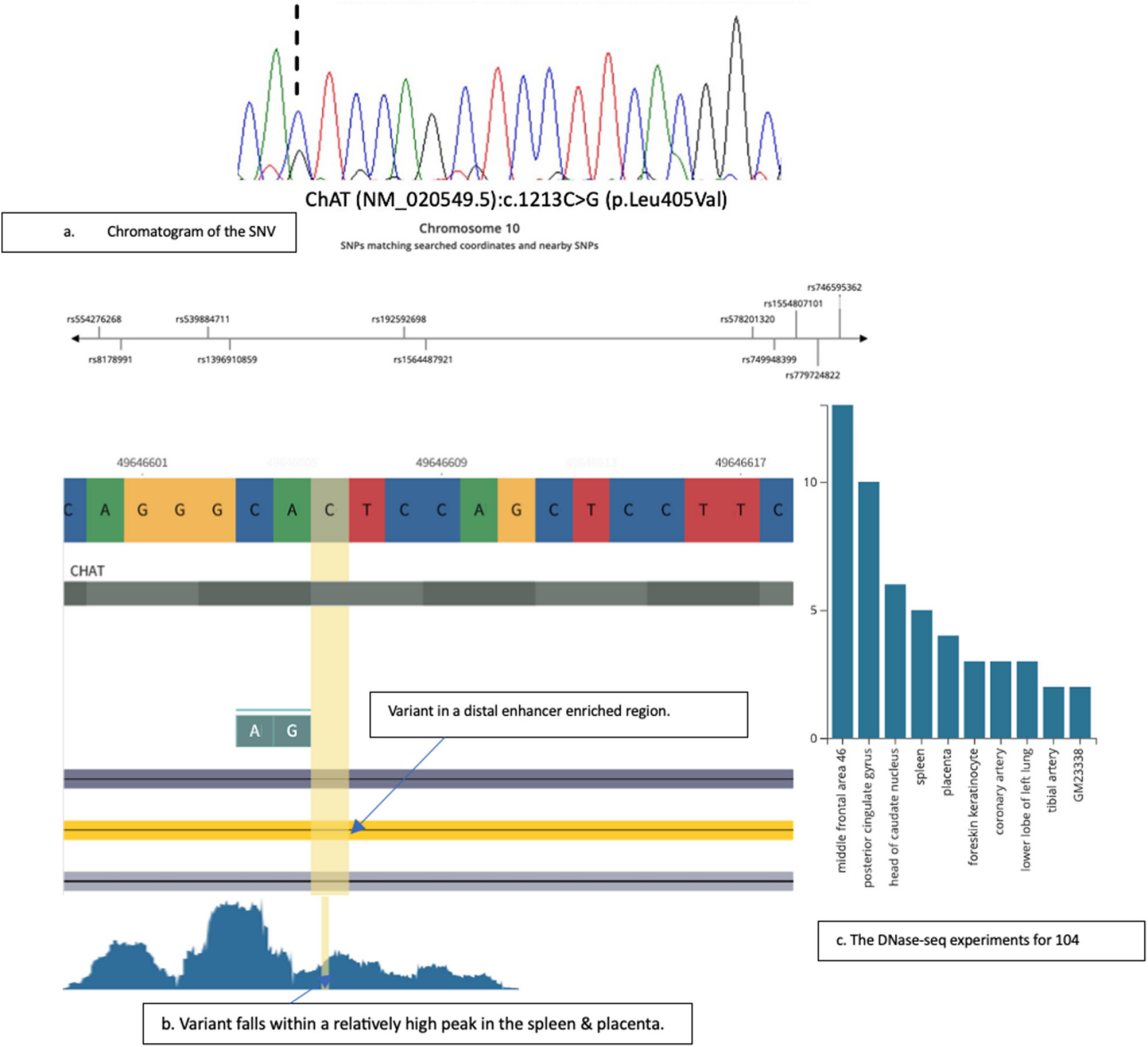
Identification and genomic context of the novel ChAT variant c.1213C>G. (a) Sanger sequencing chromatogram showing the heterozygous C>G variant (NM_020549.5:c.1213C>G) identified in the GDM patient compared to the reference consensus sequence. The arrow indicates the position of the variant. (b) Genomic view of the variant locus annotated with RegulomeDB data, indicating the variant’s position within a predicted distal enhancer region exhibiting repressive chromatin marks. (c) DNase-seq tracks displaying chromatin accessibility (peaks) at the variant locus across multiple tissues. Signals indicative of open chromatin are highlighted in the spleen and the placenta.

The analyses showed that none of the disease-causing pathogenic missense variants in the *ChAT* gene (Table S2) were identified in the patients and controls. Also, no SNP was identified with the consensus sequences in the women with GDM and controls. In a single patient, we identified a novel heterozygous missense variant in exon 8 of the *ChAT* gene. The variant *ChAT* (NM_020549.5): 10-49646606-C-G is absent in the other GDM patients or controls (Figure 1). The variant occurred in a region and around tissues that could affect the gene functions. Table 1 describes the variant annotation results in detail. *In silico* analyses of this variant *ChAT* (NM_020549.5): c.1213C>G (p. Leu405Val) suggest a variant of uncertain significance with a phyloP conservation score of 0.63. The Varsome score for this variant is 0.29 (missense variant). The variant altered the protein consensus sequence at position 405 by changing leucine to valine. When this variant was analysed in gnomAD SVs v2.1 (controls), no variant was reported in this position (Chr 10: 49646606 C-G). When we explored the closest bases to this position, two synonymous variants, and p. Ala404Val, also a missense variant of uncertain significance, as well as a stop gain variant at p.Gly411Ter, are the closest rare variants to the novel variant identified in this study (Figure 1).

**Table 1:** Annotation and *in silico* prediction scores for the *ChAT* variant c.1213C>G (p. Leu405Val)

Category	Result
Location	Exonic
Exon number	8
Type	Nonsynonymous SNV
ClinGen haploinsufficiency	10 (autosomal recessive, het; haploinsufficient)
CADD phred	16.7
Mutation taster	0.98
Phylop conservation	0.418
Epigenetic role	Active (repressor and transcription activity)
gnomAD AF	0
gnomAD mutation rate	0.059
aPC-protein function score	21.42 (high)
AlphaMissense	0.10
RegulomeDB score	0.609

**Figure 2:**
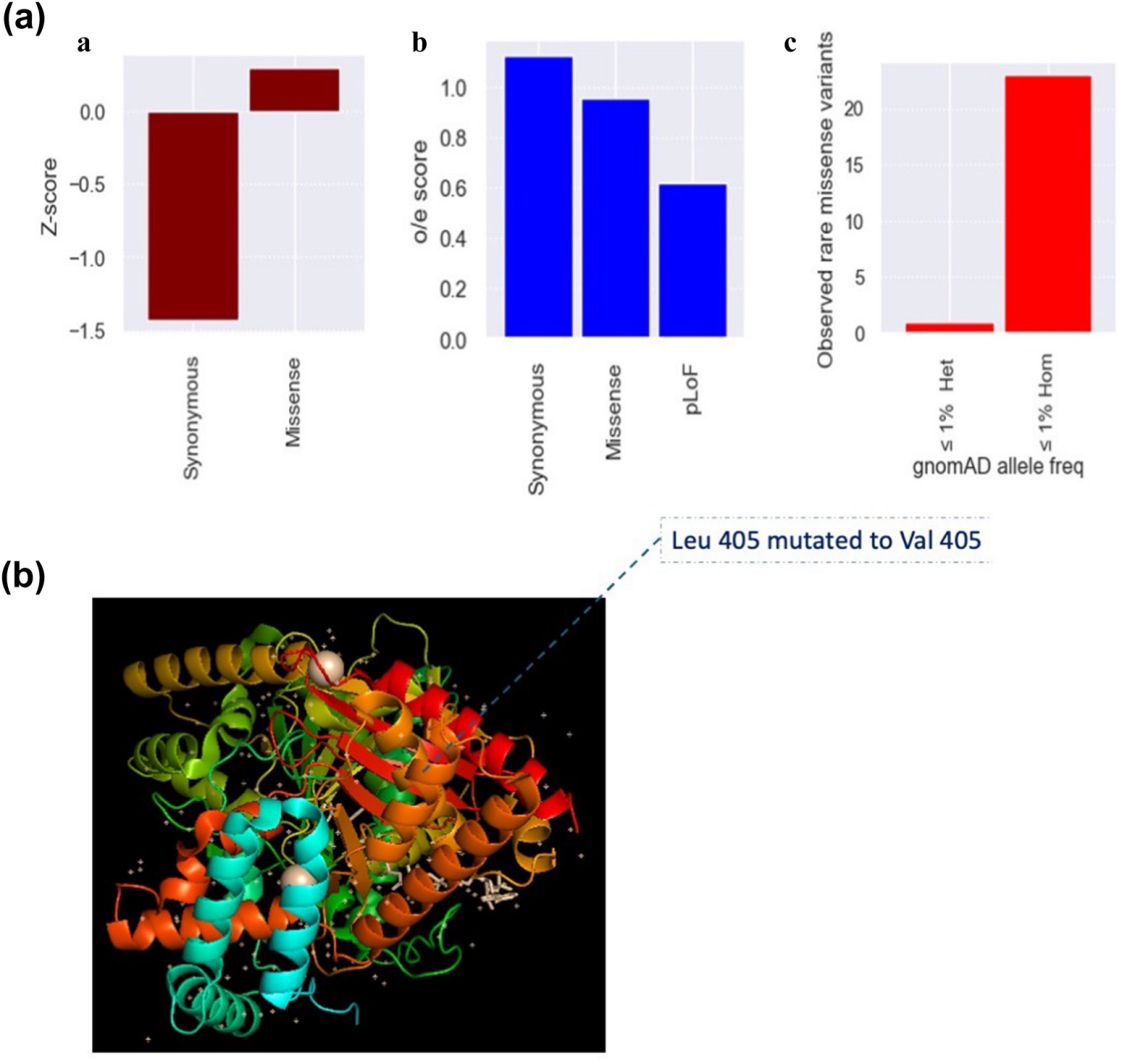
Intolerance to variation and structural details of the *ChAT* p. Leu405Val variant. (a) Gene constraint analysis for *ChAT* using gnomAD data. Plots show metrics like pLI, missense *Z*-score, and LoF o/e ratio, illustrating intolerance to variation. The analysis reflects the observed low frequency of rare (MAF ≤ 1%) and ultra-rare (MAF ≤ 0.5%) missense alleles in the population. (b) A structural model of human ChAT (from PDB ID: 7AMD) focusing on the p. Leu405Val variant site. The image displays the substitution of leucine (wild-type, B-factor = 7.90) with valine (variant, B-factor = 7.95) at position 405. Both residues are non-polar.

The extended analyses focused on the *in silico* gene functions and the effect of genetic variations. The ClinGen haploinsufficiency score for *ChAT* is 10 (Table 1), which implies that it is haploinsufficient. This finding connotes that the heterozygous variant identified in this study could be affecting the *ChAT* gene function in the patient, which the gnomAD *Z*-score, o/e score, and pLI score also support. The result implies that the *ChAT* gene is intolerant to loss-of-function (LoF) and missense variants (Figure 2a). In addition, based on the gnomAD population data, the *ChAT* gene rarely has rare (MAF ≤ 1%) and ultra-rare (≤0.5%) missense heterozygous co-occurrence variants, which underlined why only one heterozygous variant was identified in the same strand in this study.

Subsequently, we performed protein structural modelling to predict the direct impact of the p. Leu405Val substitution. Using the human ChAT protein crystal structure (PDB ID: 7AMD) template (very high [pLDDT > 90]), the Leu-to-Val change at position 405 was introduced *in silico* and analysed using PyMOL. ChAT is a monomeric enzyme composed of two domains that form an interfacial active site tunnel. The enzyme catalyses the transfer of an acetyl group from acetyl-CoA to choline, producing the neurotransmitter ACh. The active site is strategically positioned to minimise the distance between the acetyl group donor and the choline acceptor, facilitating efficient catalysis. This analysis revealed no predicted significant disruption to the protein’s three-dimensional structure. Both leucine and valine are non-polar (uncharged), hydrophobic amino acids, maintaining the local chemical environment within a protein predominantly composed of non-charged residues (∼95.9%). While a minimal increase in the calculated B-factor (atomic displacement parameter) was noted for Val405 (7.95) compared to Leu405 (7.90), suggesting a slight potential increase in local residue flexibility, this difference is minor. A positional ranking value (2847/4500), further detailed in Figure 2b, was also derived for this residue. Therefore, despite ChAT’s established intolerance to variation at the gene level, computational modelling of the specific p. Leu405Val variant did not predict a substantial structural consequence.

**Figure 3:**
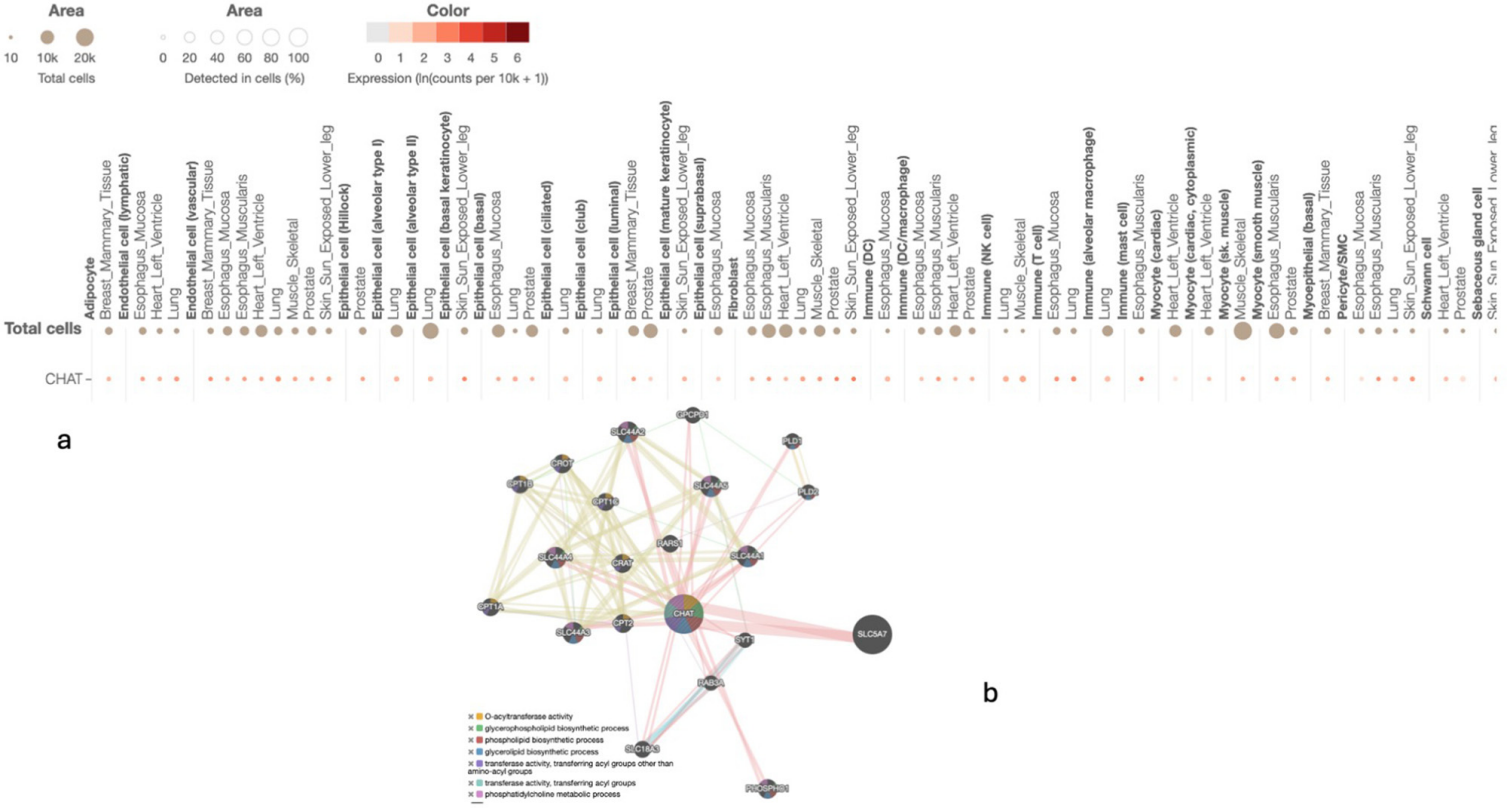
Single-cell expression profile of *ChAT* and its protein interaction network. (a) Single-cell RNA sequencing expression profile of the *ChAT* gene across various cell types, derived from GTEx data. Data are displayed in a matrix or dot plot format, where visual features (e.g., dot colour, dot size) represent the median expression level in expressing cells and the fraction of cells with detectable *ChAT* transcripts, respectively. (b) The protein–protein interaction network is centred on ChAT and generated using GeneMania. Nodes represent proteins, and edges depict functional associations based on integrated genomic data. The network highlights proteins significantly interacting with ChAT, potentially annotated by top-enriched biological pathways (pathway analysis *p* < 0.0001). Key functional partners such as SLC18A3 (VAChT) and ACHE are shown within the network context [20].

On the other hand, the tissue expression of the *ChAT* gene in the placenta is RPKM 2.7. Figure 3a shows the GTEx single-cell expression of the *ChAT* gene. The statistical summaries showed the median expression value (the dot colour) and fraction of cells in which *ChAT* was detected. We observed from this data some important findings that show that the *ChAT* gene is expressed in cells like epithelial, endothelial, adipocyte, and immune cells, to buttress our points about *ChAT’s* plausible association with GDM. The protein–protein interactions of the *ChAT* protein showed co-expressed and co-regulated proteins. The significant (*p* < 0.0001) pathways analysis showed the top seven biological pathways with the proteins that interact with *ChAT* (Figure 3b). Profoundly, the *SLC18a3* protein and AChE protein were strongly functionally related among others when compared to the coverage scores across various biological pathways (Figure 3b). It informed why the *ChAT* gene has the human phenotype ontology-terms like HP:0003306 Spinal rigidity, HP:0003458, EMG: myopathic abnormalities, HP:0003324, Generalised muscle weakness, HP:0003325, Limb-girdle muscle weakness, and HP:0003388, Easy fatigability which are all similar symptoms we observed in women with GDM in this study.

## 4 Discussion

This study identified a rare pathogenic variant in the *ChAT* gene. It is suspected to have a causative or modifier effect. Modifier variants, which are genetic variations that do not directly cause disease but can alter the expression of other genes, can significantly impact disease phenotypes, leading to variations in disease severity, penetrance, and expressivity. Identifying genetic modifiers is an existing challenge, especially for rare diseases. It can be seen as a two-front challenge, experimental and computational. We used computational approaches in this study. Future research efforts in the area of high-throughput modifier identification, in combination with experimental and computational methods, will not only help with the better diagnosis of Mendelian diseases in a time-efficient manner, but they may also provide direction to their potential therapeutic modulation, which we significantly lack. It is also expected that environmental factors may play a huge role in discovering the roles of the *ChAT* gene in GDM in South Africa. Environmental context (gene–environment interactions) plays a significant role in variant penetrance and expressivity, particularly for variants associated with common diseases such as diabetes, asthma, depression, and cardiovascular disease. South Africa is highly prone to changing environmental factors as a rapidly developing country. In the future, we will evaluate the rise in the prevalence of GDM and study the genetic contributions to pathogenicity. The gene–environment interactions will affect an organism’s traits at the level of genes, cells, tissues, and whole organisms. Under the simplifying assumption that most genetic effects are due to variants acting on genes or their products, we can separate variant effects on a trait into two components: the contribution of that variant to the target gene and the impact of that gene on the trait of interest. Most pathogenicity predictors have been developed to predict the effect of missense substitutions, which are primarily caused by single-nucleotide variants (SNVs) in the protein-coding regions of the genome. And in most cases, they have turned out to be true in genotype–phenotype studies. Some also incorporate human variation and disease data by querying gene or variant-level understanding from open-source or proprietary databases. These aggregated data generate statistical prediction models, such as supervised machine learning classifiers, which produce a score used to assign pathogenicity status to a given variant.

The genetic contribution to the onset and progression of GDM is rarely investigated; hence, there is limited data on this topic. This study leveraged the understanding that ACh regulates insulin secretion by the pancreas and is crucial in glucose handling. The prominent role of *ChAT* is synthesising ACh from acetate and choline to improve choline reuptake [21,22]. GDM women are at risk of adverse outcomes such as hypertension, preeclampsia, and future T2D, as well as cognitive impairments observed in women diagnosed with GDM [2,3]; therefore, discovering novel disease-predisposing genes or novel druggable gene targets could mitigate these risks and improve clinical outcomes. Identifying a novel missense variant in the *ChAT* gene in a patient in this study is the hallmark of our findings. The data resources that store evidence-based genotype–phenotype association information are constantly increasing; to a reasonable extent, clinicians and researchers can utilise these records to interpret disease formation, progression, diagnosis, and treatment from a genetic perspective. The goal of variant annotation is to identify and prioritise variants, both newly discovered and existing, based on expected consequences and severity. The standard annotation tools used in this study efficiently denote the plausible impact of this variant on the structure and biological or biochemical functions, including identifying DNA features and regulatory elements affected by this variant. The variant prediction tools aim to accelerate the development of precision medicine, given that most of the strategies discussed above aim to estimate disease occurrence based on the assumption that changes in protein function can lead to a decrease in organismal fitness.

Not being able to identify a known disease-causing pathogenic variant in the *ChAT* gene in the patients is not a limitation to this study because, to our knowledge, this is the first time the *ChAT* gene has been studied in GDM patients. Mutations in C*h*AT genes have been strongly associated with myasthenic syndrome, while variants in *ChA*T genes have been reported in dementia, Parkinson’s disease, and psychiatric conditions [4,13,14]. The variant identified in this study could explain GDM pathogenicity in a patient without a clear genotype–phenotype correlation. Moreover, the variant is novel in the gnomAD database and not found in the ethically matched controls. Though the variant is heterozygous in an autosomal recessive gene, as seen from the ClinGen *ChAT* gene haploinsufficiency score of 10, it strongly supports haploinsufficiency, suggesting the variant’s importance to gene dosage. Gene dosage can have a severe effect on GDM during the second and third trimesters of pregnancy when glucose homeostasis is not optimal. Pregnancy is associated with a higher demand for choline intake due to accelerated one-carbon metabolism and the synthesis of new membranes as cells undergo division. Functionally, the modifier effects of these variants can be determined in an epigenetic assay, placental gene expression analysis, and *ChAT* enzyme activity assays using a mouse model and *in vitro* cell-based assays or placentas obtained from a mouse model of GDM, as well as using the cultured cells from the placenta of donors; also, the therapeutic investigation based on the initial findings in this study would leverage approaches recently described [23].

Moreover, GWAS evidence shows that the rs2237892 variation of *KCNQ1* gene is significantly associated with increased glucose levels, impaired insulin secretion, and higher GDM risk (OR: 1.99) in the Asian population. Another GWAS showed that *MTNR1B* polymorphism was associated with glycaemic levels in both gravid and non-gravid populations. The associations between SNPs rs7936247, rs4753426 in *MTNR1B* and GDM risk have also been reported. The *KCNQ1* variant is intronic, *KCNQ1*: NC_000011.10:2818520:CT. Also, *MTNR1B* NC_000011.10:92968429:T:A is a non-coding transcript variant associated with GDM. In all, they are one single base change variation. In this study, the variant *ChAT* (NM_020549.5):c.1213C>G (p.Leu405Val) is a missense singleton in a coding region. The key question is which genes and regulatory regions will most likely yield the most helpful information for precision medicine. Future studies on these genes will shed more light on the question. The current approaches to deciphering biological mechanisms of specific variants through variant-to-function analyses remain laborious and highly specialised, but are important for future direction.

GDM has been reported to cause a marked reduction of choline, suggesting that genes and receptors involved in choline synthesis and metabolism should be investigated in GDM patients with impaired cholinergic systems, and how to reduce its adverse effects. For example, in some cases of GDM, choline supplements are prescribed to prevent foetal overgrowth, altered placental morphology, macrosomia, and excessive adiposity [10,24]. If these circumstances are not prevented, they could have a significant epigenetic effect on the offspring. Similarly, the hyperglycaemia associated with GDM may cause vascular damage through oxidative stress. The release of proinflammatory biomarkers can cause vascular system damage, further impairing cholinergic activities in the brain and resulting in brain dysfunction. Resultant brain dysfunction can manifest in various phenotypes linked to stress, hormonal imbalance, neuroinflammation, and epigenetic disorders of the brain [24–27]. This finding is consistent with human studies showing that depression can be precipitated by hyperactivation of the cholinergic system [22], and what individuals ingest regularly could be a contributory factor to the ACh modulation of depression, as it was previously discovered that monosodium glutamate in food seasoning may have depressive symptoms [28]. In addition, exposure to stress can alter the function of cholinergic enzymes, including AChE [22,26,27]. A study revealed that ACh-induced vasodilatation in both hands and feet was lower in women previously diagnosed with GDM than in controls [29] and, therefore, could contribute to inter-organ communication that regulates GSIS. Moreover, several neurotransmitters released from the peripheral autonomic neurons, including Ach, have been proposed to modulate GSIS [30,31]. Furthermore, in addition to ACh, parasympathetic neuronal endings in the pancreas release many other neurotransmitters, including Kisspeptin 1, which suppresses GSIS from β cells and has been shown to cause impaired glucose tolerance in mice [32].

The *ChAT* protein is the biosynthetic enzyme for the neurotransmitter ACh in the central and peripheral nervous systems and is highly expressed in the placenta and brain https://www.proteinatlas.org/ENSG00000070748-CHAT. Notably, the human *ChAT* gene is located on chromosome 10 and has 15 coding exons and 748 amino acids (NC_000010.11 ref GRch38 Assembly). Mutations in the *ChAT* gene have been strongly associated with myasthenic syndrome, while variants in the *ChAT* gene have been reported in dementia, Parkinson’s disease, and psychiatric conditions [4,7,11]. A dysfunctional cholinergic system can affect many gene expressions, leading to multiple disorders. This study established that through the gene enrichment analyses the ChAT protein interacts with other specific functional proteins like the SLC*18A3*, a solute carrier gene. Interestingly, genetic association in this solute carrier with GDM was observed in a meta-analysis [20]. It is, therefore, essential to study the *ChAT* gene in the light of other functional pathways and genes. GeneMANIA predicts gene function from the composite network using a variation of the Gaussian field label propagation algorithm appropriate for gene function prediction. Remarkably, *ChAT* gene interaction is prominent with certain biological functions relevant to GDM pathology. Besides these pathways being implicated in the pathology of GDM, they also serve as potential therapeutic targets.

Although we used the Sanger sequencing technique in this study, WES, NGS, and Sanger sequencing remain the preferred choice for screening and validation of disease-causing or predisposing mutations and SNPs; it is a stark reality that this sequencing technology remains inaccessible to underprivileged communities. Furthermore, using cost-effective genotyping methods to genotype variants in the *ChAT* gene, as we had previously described [33], will improve rapid genetic diagnosis in GDM cases for clinical utility in low-resourced settings.

The most challenging variants are usually missense because they are abundant in the human genome, and their clinical relevance demands functional studies. Of the more than 4 million observed missense variants, only 2% have been clinically classified as pathogenic or benign. The lack of accurate missense variant functional predictions limits the diagnostic rate of rare diseases and the development or application of clinical treatments that target the underlying genetic cause. Using the missense substitution analysis algorithms in a clinical genetics context involves crossing a psychological barrier as much as a methodological one. The algorithm predictions used in this study have the potential to accelerate our understanding of the molecular effects of variants on protein function, contribute to the discovery of disease-causing genes, and increase the diagnostic yield of disorders like GDM. The novel variant identified in this study may act as a disease modifier. A modifier variant generally lessens the phenotype imparted by a causal variant and can be evaluated through the interacting genetic networks [34]. Genetic variants are major determinants of disease susceptibility, response to therapy, and clinical outcomes, some genetic variants with strong link to certain diseases could be studied in the light of *ChAT* gene which is the latest in the list of gene of interest to us, particularly to study their profound genetic effects on African samples [13,33–37]. While many variants have been linked to the onset and susceptibility to various diseases, only a few have clinical diagnoses and druggable targets [38]. Nevertheless, the approach that we used in this study is limited to the screening of a few protein-coding regions in a single gene. Also, the sample size in this study is small compared to other studies from Asia. Nonetheless, little is known about the genetic contribution to GDM globally, with almost no data in Africa. As knowledge develops in this area in Africa, we can replicate a comprehensive ontology database and genetics research capacity for GDM in Africa, like the hearing impairment in Africa consortium [39,40].

The major limitation of this study is the very limited sample size. Nonetheless, since the primary objective for this study was to explore causal variants in a single gene, a 1:1 or 2:1 case–control sample size could be adequate but if we were to analyse multiple genetic markers, a larger sample size is a necessity.

In conclusion, understanding the genetic basis of a disease is a continuous effort because genetic diseases often evolve with new mutations. Future work will entail performing whole-exome or whole-genome sequencing on the same sample with validation in a larger sample size to acquire robust genetic data that could detect novel variants with causal portfolio in GDM in these patients and African cohorts, respectively. We also advise other researchers interested in GDM to implement a targeted gene sequencing approach comprising the *ChAT* gene and other ACh-regulated genes, like the solute carrier genes, e.g., SLC18A3, found to be associated with the *ChAT* gene in the network analyses in this study.

## Supplementary Material

Supplementary Table
