# Supplementary material for: The identification of novel missense variant in ChAT gene in a patient with gestational diabetes denotes plausible genetic association
Source: Open Med (Wars). 2025 Jul 17;20(1):20251225. doi: 10.1515/med-2025-1225 (PMC12273656; doi:10.1515/med-2025-1225)
Supplement: Supplementary Table [file med-2025-1225-sm.pdf]

Supplementary material

Table S1: Nanodrop® DNA quantification results for samples and control

DNA sample	DNA quantification (ng/µl)
GDM 2	259
GDM 10	397.7
GDM 42	477.7
GDM 3	148.1
GDM 26	87.21
GDM 29	186.8
GDM 39	148.3
GDM 1	68.55
GDM 22	236.5
GDM 34	185.3
GDM 36	98.2
GDM 41	134
ND 20	282.3
ND 32	144.8
ND 50	167.0
ND 8	38.36
ND 15	13.85
ND 22	110.8
ND 30	146.7
ND 14	91.63
ND 34	122.5
ND 40	123

Table S2: Sequencing quality report for each sample sequenced both forward and reverse reactions

Sample file name	Sample name	Well ID	Cap#	Median PUP	Trace score	CRL	Signal strength
E9_E9_20220208_184745.ab1	E9	E9	1	0	22	47	117.75
F9_F9_20220208_184746.ab1	F9	F9	2	0	23	74	179.5
G9_G9_20220208_184747.ab1	G9	G9	3	0	23	72	176
H9_H9_20220208_184748.ab1	H9	H9	4	0	24	71	97.25
A9_A9_20220208_181851.ab1	A9	A9	1	0	14	27	252
B9_B9_20220208_181852.ab1	B9	B9	2	0	16	28	166.25
C9_C9_20220208_181853.ab1	C9	C9	3	0	14	0	56.5
D9_D9_20220208_181854.ab1	D9	D9	4	0	15	22	562.25
E8_E8_20220208_174954.ab1	E8	E8	1	0	14	36	90
F8_F8_20220208_174955.ab1	F8	F8	2	0	13	20	239.5
G8_G8_20220208_174956.ab1	G8	G8	3	0	13	13	86.75
H8_H8_20220208_174957.ab1	H8	H8	4	0	13	11	73.75
A8_A8_20220208_172100.ab1	A8	A8	1	0	16	40	88.5
B8_B8_20220208_172101.ab1	B8	B8	2	0	14	1	41
C8_C8_20220208_172102.ab1	C8	C8	3	0	16	32	286.5
D8_D8_20220208_172103.ab1	D8	D8	4	0	14	25	879.75
E7_E7_20220208_165206.ab1	E7	E7	1	0	12	18	69.75
F7_F7_20220208_165207.ab1	F7	F7	2	0	12	5	624.75
G7_G7_20220208_165208.ab1	G7	G7	3	0	15	29	109.75
H7_H7_20220208_165209.ab1	H7	H7	4	0	15	21	99
A7_A7_20220208_162312.ab1	A7	A7	1	0	12	0	114
B7_B7_20220208_162313.ab1	B7	B7	2	0	11	0	55.75
C7_C7_20220208_162314.ab1	C7	C7	3	0	13	19	135.25
D7_D7_20220208_162315.ab1	D7	D7	4	0	13	10	556.5
E6_E6_20220208_155418.ab1	E6	E6	1	0	11	0	71.5
F6_F6_20220208_155419.ab1	F6	F6	2	0	0	0	11
G6_G6_20220208_155420.ab1	G6	G6	3	0	10	0	51.5
H6_H6_20220208_155421.ab1	H6	H6	4	0	13	0	43.25
A6_A6_20220208_152525.ab1	A6	A6	1	0	12	0	80.75
B6_B6_20220208_152526.ab1	B6	B6	2	0	12	5	87.25
C6_C6_20220208_152527.ab1	C6	C6	3	0	12	0	56.5
D6_D6_20220208_152528.ab1	D6	D6	4	0	11	0	53.25
E5_E5_20220208_145630.ab1	E5	E5	1	0	7	0	33.25
F5_F5_20220208_145631.ab1	F5	F5	2	0	11	0	33.5
G5_G5_20220208_145632.ab1	G5	G5	3	0	10	0	26.25
H5_H5_20220208_145633.ab1	H5	H5	4	0	8	0	29.25
A5_A5_20220208_142734.ab1	A5	A5	1	0	8	0	26
B5_B5_20220208_142735.ab1	B5	B5	2	0	11	0	104.5
C5_C5_20220208_142736.ab1	C5	C5	3	0	9	0	69.75
D5_D5_20220208_142737.ab1	D5	D5	4	0	10	0	57.75

Table S3: Disease-associated pathogenic missense variants in the ChAT gene obtained from ClinVar and OMIM databases

Canonical Position	Ref allele	Alt allele	dbSNP ID	Conditions	In OMIM	MOI
49627734	C	T	rs769234940	Myasthenia	No	
49646654	G	C	—	Myasthenia		
49649511	C	T	—	Myasthenia		
49649532	C	T	—	Myasthenia		
49649617	C	T	rs121912821	Myasthenia	Yes	AR
49649634	C	T	—	Myasthenia		
49651887	G	T	rs121912817	Myasthenia	Yes	AR
49655101	C	T	rs369251527	Myasthenia	No	
49655140	C	T	—	Myasthenia		
49662695	G	A	—	Myasthenia		
49662713	G	T	—	Myasthenia		
49664879	C	G	rs201439531	Apnea, pneumonia	No	
49619754	C	T	rs794727516	Myasthenia	No	
49619808	C	T	—	Myasthenia		
49620543	T	C	rs121912820	Myasthenia	Yes	AR
49620545	C	G	rs121912815	Myasthenia	Yes	AR
49614273	A	T	rs772025588	Myasthenia	No	
49625633	T	C	rs75466054	Myasthenia	No	

MOI = mode of inheritance, AR = Autosomal recessive.
